# Epidemiological and Clinical-Pathological Aspects of *Helicobacter pylori* Infection in Brazilian Children and Adults

**DOI:** 10.1155/2018/8454125

**Published:** 2018-09-04

**Authors:** Evelyn Pedroso Toscano, Fernanda Fernandes Madeira, Mayra Pinheiro Dutra-Rulli, Luiz Otavio Maia Gonçalves, Marcela Alcântara Proença, Viviane Silva Borghi, Aline Cristina Targa Cadamuro, Giselda Warick Mazzale, Ricardo Acayaba, Ana Elizabete Silva

**Affiliations:** ^1^UNESP-São Paulo State University, Department of Biology, São José do Rio Preto, SP, Brazil; ^2^UFMS-Federal University of Mato Grosso do Sul, School of Medicine, MS, Brazil; ^3^João Paulo II Hospital, Service of Endoscopy, São José do Rio Preto, SP, Brazil

## Abstract

**Aim:**

To evaluate the prevalence and risk factors of *H. pylori* infection in the pediatric and adult population seen at a public hospital in São José do Rio Preto, SP, Brazil.

**Methods:**

This is a retrospective study that evaluated 2406 medical records of children, adolescents, and adults with dyspeptic symptoms who underwent upper gastrointestinal endoscopy. *H. pylori* diagnosis and demographic and clinical-pathological features were recorded.

**Results:**

A total of 852 subjects were *H. pylori* positive, with an overall prevalence of infection of 35.4%, occurring mainly in adults over 40 years of age, and a 24.7% prevalence considering only children and adolescents. No association was observed between *H. pylori* infection and risk factors. However, the *H. pylori* positive individuals showed a higher frequency of pangastritis (*p* < 0.01), severe lesions (*p* = <0.001), and erosive lesions (*p* = 0.04). The bacterium was eradicated in 83.5% (127) of the patients who received the standard therapy.

**Conclusions:**

The prevalence of *H. pylori* detected in a public service in São José do Rio Preto, SP, Brazil, is as expected for developed countries, showing growing rates with increasing age. As *H. pylori* infection occurs during childhood, screening programs for detection and prevention in the pediatric population are important to reduce the prevalence of this infection in adults.

## 1. Introduction

The infection by *Helicobacter pylori* (*H. pylori*), a gram-negative bacterium, is among the most frequent infections worldwide, affecting about 50% of the global population and an estimated 4.4 billion individuals likely to be infected in 2015 worldwide [1].

*H. pylori* infection causes chronic inflammation and is the main etiologic agent associated with chronic gastritis, peptic and duodenal ulcer, MALT (mucosa-associated lymphoid tissue) lymphoma, and gastric adenocarcinoma [2]. Having been recognized as a risk factor for stomach cancer, *H. pylori* was classified by the International Agency for Research on Cancer [3] as a class I carcinogen, which was involved in 90% of all gastric malignancies [4]. The clinical consequences of *H. pylori* infection are determined by multiple factors, including genetic predisposition of the host, especially certain cytokine polymorphisms, environmental factors, such as high dietary salt intake, smoking, and drinking, besides heterogeneity of *H. pylori* strains [5].

The bacterial virulence factors such as cagA (cytotoxin-associated gene A) and its pathogenicity island (cag PAI) and vacA (vacuolating cytotoxin A) are associated with more severe inflammation of the gastric mucosa, conferring a greater risk of developing gastric carcinoma and a poorer clinical outcome of the infection [6, 7]. In general, it is observed that colonization by the bacteria causes a severe systemic immune response, inducing the production of proinflammatory cytokines, reactive oxygen species (ROS), and reactive nitrogen species (RNS), activation of NF-*κ*B (transcription factor nuclear factor), cellular proliferation, and survival factors [6, 7]. All these changes can alter cellular homeostasis and trigger the malignant progression cascade to gastric cancer, such as chronic gastritis, atrophic gastritis, intestinal metaplasia, dysplasia, and gastric adenocarcinoma [8].

The gastric cancer incidence and mortality rates remain globally high, especially in Asia and Eastern Europe and also in developing countries of South America [5]. It is the fifth most common cancer type worldwide and the third leading cause of cancer-related death, occurring mainly in China, Japan, and Korea [1, 9]. In Brazil, it ranks fourth in incidence and second in death rate [10].

*H. pylori* is often acquired in childhood due to fecal-oral and oral-oral transmission and is associated with socioeconomic conditions, particularly sanitary conditions, socio-demographic subgroups, high household density, low education levels, and rural residence [1, 11].

Although most infected individuals are asymptomatic, if not treated, *H. pylori* infection may persist for decades in its hosts and promote the development of gastrointestinal diseases [12]. Thus, *H. pylori* eradication is considered a first-line treatment to achieve the eventual regression of acute and chronic inflammation and to prevent malignant progression [5]. Considering that a reduction in the prevalence of *H. pylori* can contribute significantly to a decline in the incidence of gastric cancer, a better understanding of its epidemiology may help developing prevention and intervention programs for this infection, which in turn will result in a decrease of the incidence of this neoplasm.

Brazil is a very large country, with great regional differences as to the quality of life and health. Some epidemiologic studies have reported great variation in the prevalence of *H. pylori* according to the region investigated, showing a higher prevalence in the north of the country compared to the south [13–16]. The aim of this study was to determine the prevalence of *H. pylori* infection and risk factors in pediatric and adult patients seen at a public hospital in São José do Rio Preto, SP, Brazil. Hospital-based studies are important to predict population estimates and establish programs for the prevention and control, information for population, and treatment of *H. pylori* infection.

## 2. Materials and Methods

This study was approved by the Research Ethics Committee of IBILCE/UNESP (protocol 44384).

This is a retrospective study that evaluated 2406 medical records of children, adolescents, and adults with dyspeptic symptoms who underwent examination by upper gastrointestinal endoscopy (UGE) from May to December 2009 and from January to July 2012 at the public Hospital João Paulo II in São José do Rio Preto, northern region of the State of São Paulo (SP), Brazil. *H. pylori* infection was assessed by the rapid urease A test.

Demographic and clinical-pathological features such as age, gender, place of residence (rural or urban), *H. pylori* diagnosis, endoscopy diagnosis according to the Sydney system [17], therapy for *H. pylori* infection eradication, alcohol consumption (nondrinkers or social consumption x everyday drinkers), and smoking (current smoker or former smoker x non-smoker) were collected from the medical records. The standard triple therapy for bacterium eradication consisted of amoxicillin (1 g), clarithromycin (500 mg), and omeprazole (20 mg), all twice daily for seven days. All this information was stored in an Excel database, using codes without identifying the individuals.

### 2.1. Statistical Analysis

Descriptive analysis was used for calculating the mean age and other parameters evaluated. Fisher's exact test was also used to determine the association between *H. pylori* infection and the parameters gender, age, residence, alcohol consumption, smoking habits, treatment, and eradication. Mean values were compared using the *T* test. Statistical analysis with a significance level of *p* < 0.05 was performed using GraphPad Prism software v.6.01.

## 3. Results

Table 1 shows the distribution of demographic and clinical-pathological characteristics of subjects with dyspepsia who underwent UGE. In the 2406 medical records evaluated, the prevalence of *H. pylori* infection considering all age groups was 35.4% (852). The mean age of *H. pylori* positive (Hp+) and *H. pylori* negative (Hp−) individuals was similar (47 ± 15 years versus 48 ± 17 years). However, when the subjects were stratified by age group, there was a higher prevalence of *H. pylori* infection in adults between 40 and 60 years (*p* < 0.001).

Regarding gender, there was a higher frequency of females in both groups Hp+ (65.7%) and Hp− (68.1%) compared to males (34.3% and 31.9%, respectively). In both groups, most individuals lived in urban homes (Hp+ = 92.0%; Hp− = 92.7%), did not smoke (Hp+ = 63.4%; Hp− = 62.7%) nor drink (Hp+ = 70.9%; Hp− = 74.2%), without any significant difference between the infected and the uninfected groups.

Of the 852 Hp+ individuals, the medical records contained information about the bacterial eradication therapy for 245, of which 80% (196) claimed to have correctly carried out the proposed treatment. Of the 196 subjects who followed the treatment, 83.5% (127) had the bacterium eradicated, as confirmed by a negative result in a second endoscopy examination.

Considering the individuals who underwent UGE, only 1.8% (15) of the Hp+ group versus 2.5% (39) of the Hp− group had a normal stomach according to the endoscopy diagnosis, while most had some type of gastric lesion (Hp+ = 98.2% versus Hp− = 97.5%). In both groups, the most common lesions diagnosed according to the Sydney system were gastritis and pangastritis, besides other less frequent gastric lesions such as polyps and peptic ulcers; intestinal metaplasia was observed in one subject of the Hp+ group.

Furthermore, when we evaluated the endoscopic gastritis classification in the Hp+ versus Hp− groups according to its location, intensity, and category (Table 2), we observed significant differences between the groups, due to a higher prevalence of pangastritis (Hp+ = 48.8% vs Hp− = 39.4%, *p* < 0.01) and severe lesions in the Hp+ group (Hp+ = 12.9% versus Hp− = 6.9%, *p* < 0.01). Regarding the lesion category, the Hp+ group had a higher frequency of erosive lesions (51.9%) while the Hp− group showed more enanthematous lesions (54%) (*p* = 0.04).

In another analysis, we analyzed only children and adolescents aged less than or 18 years, corresponding to 3.2% (77/2406) of all assessed medical records (Table 3). In this group, only 19 individuals were Hp+, with a prevalence of 24.7% of bacterial infection. The mean age in the Hp+ group (16.2 ± 1.9 years, range 10–18years) was similar to that of the uninfected group (15.2 ± 2.3 years, range 9–18 years, *p* = 0.12). Regarding gender, the prevalence of females was higher among both infected (89.5%) and uninfected subjects (75.9%). Most of them (94.7% of Hp+ and 89.1% of Hp−) were of urban origin. In the Hp+ pediatric group, endoscopy showed 100% of individuals with endoscopic lesions, while in the Hp− group 97.5% (51) it showed some type of gastric lesion, and 2.5% (7) of them had a normal mucosa. As to the endoscopic classification of gastritis (Table 4), the Hp+ pediatric individuals showed mainly pangastritis (66.7%) followed by antral gastritis (33.3%), while the Hp− individuals had mostly antral gastritis (58.8%) followed by pangastritis (41.2%). Regarding the intensity of the lesions, while the Hp+ pediatric individuals showed mainly moderate lesions, the Hp− subjects had mostly mild lesions; however, the differences between the groups were not significant. Regarding the category, both groups showed mainly enanthematous gastritis.

## 4. Discussion

Our study evaluated clinical-epidemiological features of *H. pylori* infection in both the adult and pediatric population of the Brazilian Southeast region, in a city of the São Paulo State inland. The overall infection prevalence was 35.4%, occurring mainly in adults over the age of 40 years. We did not detect any significant differences between the infected and the noninfected groups regarding gender, place of residence, tobacco smoking, alcohol intake, and endoscopic diagnosis. However, the *H. pylori* positive individuals showed a higher frequency of pangastritis, lesions of severe intensity, and erosive lesions.

*H. pylori* prevalence estimates show a great variation globally, with rates varying from 15.1% in developed countries (Australia) to 87.7% in developing countries (Nigeria) [1, 18]. In addition, there are great regional variations within the same country [1, 19]. For 2015, it was estimated that approximately 4.4 billion individuals were infected by *H pylori* worldwide [1]. However, in the last decades, due to improvements in sanitation, health, socio-cultural conditions, and eradication methods, a decline of the *H. pylori* infection prevalence has been observed in different countries, concomitant with a decrease of the peptic ulcer disease and gastric cancer [1, 18]. These findings reinforce the idea that bacterial eradication in areas of high gastric cancer prevalence reduces the risk of this neoplasm [2]. Our results are within the range reported for developed countries of North America (37.1%) and Europe (34.3%) [1].

In Brazil, epidemiological studies to estimate the *H. pylori* prevalence among children and adults have shown different rates according to the regions and time periods assessed. In the Northeast and Midwestern regions, higher prevalence rates are reported (63%–96%) [13, 14]. In contrast, in the Southeastern region, lower rates are reported, as for São Paulo (SP) 76.3% and Rio de Janeiro (RJ) 59.5% [13]. However, in the cities of the São Paulo State inland, elevated frequencies, over 85%, have been detected [13]. In contrast, recently Frugis et al. [20] observed a significantly decreased *H. pylori* prevalence in a private healthcare service of São Paulo, going from 19.3% in 2004 to 14.1% in 2014, concomitantly with improvements in health and socio-cultural conditions. In line with these findings, our results show a low prevalence rate of *H. pylori* infection in the region of São José do Rio Preto, as compared to other cities in Brazil and other countries (Table 5).

São José do Rio Preto is located in the Northwestern part of the State of São Paulo and is the State's 12^th^ biggest city. Its HDI (Human Development Index) was 0.797 in 2010, as measured by the IBGE (Brazilian Institute of Geography and Statistics), an index similar to Rio de Janeiro (0.799) and São Paulo (0.805) [21]. This index shows very good living conditions and health quality, with demographic and social indicators similar to developed cities.

Considering the relevance of *H. pylori* infection in childhood, we also evaluated children and adolescents. In this group the prevalence of the bacterium was lower, i.e., 24.7%. Another study, conducted in a public hospital in São Paulo, detected a seroprevalence of 35.6% in children, which increased with age, reaching 58.3% in those aged over 12 years [15] However, in children aged 4–11 years from Salvador (BA), the seroprevalence of *H. pylori* was found to be 28.7% [22], similar to our study. In the Salvador city study, the authors also observed that *H. pylori* infection was associated with increasing age and variables indicative of poor hygiene and crowded living conditions. Queiróz et al. [23] compared the prevalence of *H. pylori* infection in children of Belo Horizonte (Brazil), Santiago (Chile), and London (UK) and observed a higher prevalence in Latin America (35%) than in the UK (7%). In contrast, a review made in the city of Porto (Portugal) showed high prevalence of infection in children between 4-5 years (26%) and in adolescents (66%), with rates increasing to 73.9% between 18–30 years and exceeding 88% in individuals over 40 years of age [24]. These findings are consistent with recent estimates showing that Portugal has higher rates of *H. pylori* infection (86.4%) [1]. On the other hand, although Africa also shows a high prevalence rate (79.1%) [1], recently Awuku et al. [25] observed a low prevalence (14.2%) of *H. pylori* infection among children in a rural environment in Africa, associated with increasing household numbers, female gender, source of drinking water other than pipe and borehole, open-air defecation, and younger age. These findings corroborate that the prevalence of *H. pylori* infection is highly variable in different regions and related to socioeconomic status and hygiene and sanitary conditions. Thus, improvements in these factors should contribute to the prevention of *H. pylori* infection in childhood and consequently in adults.

Regarding clinical and pathological factors associated to *H. pylori* prevalence, we did not detect significant differences in gender, smoking and drinking habits, or other features such as place of residence and endoscopic diagnosis between the Hp positive and Hp negative groups. However, we observed that the prevalence of *H. pylori* increases with age, as reported by other studies [15, 22, 24, 26]. Regarding gender, some studies show that the infection by *H. pylori* is virtually the same in both genders [19, 27, 28], as found in the present study, but it can be positively associated with alcohol consumption [19, 28].

When we evaluated the endoscopy classification of gastritis considering all age groups, in both infected and noninfected individuals, the *H. pylori* positive patients showed mainly pangastritis of moderate or severe intensity and of the erosive category, whereas in the *H. pylori* negative individuals enanthematous antral gastritis of moderate or mild intensity was more frequent. All the *H. pylori* positive children and adolescents showed endoscopic lesions, more frequently pangastritis of moderate intensity. In a community of northern Canada, Cheung et al. [28] also detected a high prevalence of gastric lesions among *H. pylori* positive individuals, mainly chronic gastritis (100%), acute gastritis (94%), gastric atrophy (21%), and intestinal metaplasia (11%) with inflammation of moderate (47%) and severe (47%) intensity. In a pediatric population, Queiróz et al. [23] reported an association between *H. pylori* infection with gastric endoscopy findings such as the presence of antral and corpus erythema and antral nodularity, as well as with the degree of antral and corpus active and chronic inflammation in the *H. pylori* positive children.

Another important aspect of our study was the finding of an 83.5% eradication rate after *H. pylori* therapy, confirmed by a second endoscopy after treatment. This value is below the recommended efficiency rate, supposed to achieve ≥90% eradication [29]. However, the cure rate after standard triple therapy plus bismuth as a first-line treatment for *H. pylori* infection has shown great variation globally: 55–94% [29]. In Latin America, after initial treatment, a mean eradication rate of 72.2% (ranging from 30.2% to 100%) has been observed. In Brazil, the eradication success rate ranges from 60.4% to 100% [30]. It should, however, be pointed out that the eradication rate has decreased over time to below 80%, due mainly to multiple antibiotic resistance and patient compliance [12, 31, 32]. According to Gisbert and MacNicholl [29], scoring over 85% eradication rate may require two or more optimization strategies. The standard therapy employed in the patients treated in the present study consisted of the classical triple regimen composed of clarithromycin, amoxicillin, and a proton-pump inhibitor for seven days. However, quadruple therapy in combination with bismuth compounds or sequential/concomitant therapy has been recommended by European guidelines for a longer period of time (14 days) for high-resistance individuals [29, 33]. Prolongation of treatment to 14 days improved the eradication success rates in patients with resistant strains, increasing the cure rates. Moreover, the concomitant therapy has been found to be more effective than the standard triple and sequential therapy for *H. pylori* eradication [29]. This data reinforces the importance of *H. pylori* treatment and eradication, leading to reversion of acute and chronic inflammation and possibly to reversion of preneoplastic lesions and prevention of malignant progression [12, 31].

A limiting factor of our study is that it was performed in a sample of patients with dyspeptic symptoms who underwent upper gastrointestinal endoscopy, thus a hospital-based study, which may not reflect the pattern in the general population. Therefore, it is an important further community-based study with a large number of individuals to obtain more accurate data on the prevalence of *H. pylori* in the general population.

In conclusion, our data show that the prevalence of *H. pylori* detected in a public healthcare service in São José do Rio Preto (SP) is similar to that expected for developed countries, showing growing rates with increasing age. Moreover, considering that the infection occurs in childhood, screening programs for detection and prevention in the pediatric population are important to reduce the prevalence of *H. pylori* infection in adults. Thus, further studies are needed, to cover a larger geographic extension in Brazil, since the bacterial infection is widely distributed. They should also allow a better understanding of its epidemiology by analyzing the various environmental and socioeconomic conditions likely to influence its prevalence in each population group.

## Figures and Tables

**Table 1:** Distribution of demographic and clinical-pathological characteristics of subjects evaluated for *H. pylori* infection.

Variables	*H. pylori* status		Total	*P* value
	Negative	Positive		
*N (%)*	1554 (64.6)	852 (35.4)	2406	
*Age (years)*				
≤19	69 (4.4)	22 (2.6)	91	<**0.001**^1^
20–29	184 (11.9)	95 (11.1)	279	
30–39	248 (16.0)	166 (19.5)	414	
40–49	305 (19.6)	203 (23.9)	508	
50–59	334 (21.5)	187 (21.9)	521	
>60	414 (26.6)	179 (21.0)	593	
Mean ± SD	48 ± 17	47 ± 15		**0.06** ^2^
Range	9–95	10–88		
*Gender*				
Female	1058 (68.1)	560 (65.7)	1618	0.26
Male	496 (31.9)	292 (34.3)	788	
*Residence*				
Rural	109 (7.3)	65 (8.0)	174	0.59
Urban	1388 (92.7)	748 (92.0)	2136	
*Tobacco smoking*				
Yes	130 (37.3)	86 (36.6)	216	0.93
No	219 (62.7)	149 (63.4)	368	
*Alcoholism*				
Yes	78 (25.8)	59 (29.1)	137	0.47
No	224 (74.2)	144 (70.9)	368	
*Endoscopic diagnosis*				
Normal stomach	39 (2.5)	15 (1.8)	54	0.84
Endoscopic lesion	1515 (97.5)	837 (98.2)	2352	
*Eradication therapy*				
Yes	—	196 (80.0)	196	—
No	—	49 (20.0)	49	
*After therapy*				
Eradicated	—	127 (83.5)	127	—
Not eradicated	—	25 (16.5)	25	

*N* = sample number (subjects without information in the medical records were excluded from the analysis). ^1^chi-square test used to compare age categories. ^2^*t* test used to compare means of age.

**Table 2:** Endoscopy classification of gastritis according to location, intensity, and category in *H. pylori*− and *H. pylori*+ subjects.

Classification	*H. pylori* status		Total	*P* value
	Negative	Positive		
*N (%)*	1515 (64.4)	837 (35.6)	2352	
*Location*				
Antral	536 (59.6)	230 (50.1)	766	<**0.01**
Body	9 (1.0)	5 (1.1)	14	
Pangastritis	354 (39.4)	224 (48.8)	578	
*Intensity*				
Mild	310 (29.7)	118 (20.0)	428	<**0.001**
Moderate	663 (63.4)	396 (67.1)	1059	
Severe	72 (6.9)	76 (12.9)	148	
*Category*				
Enanthematous	652 (54.0)	332 (47.8)	984	**0.04**
Erosive	553 (45.8)	360 (51.9)	913	
Atrophic	3 (0.2)	2 (0.3)	5	

*N* = sample number (subjects without information in the medical records were excluded from the analysis).

**Table 3:** Distribution of demographic and clinical-pathological characteristics of children and adolescents evaluated for *H. pylori* infection.

Variables	*H. pylori* status		Total	*P* value
	Negative	Positive		
*N (%)*	58 (75.3)	19 (24.7)	77	
*Age (years)* mean ± SD	15.2 ± 2.3	16.2 ± 1.9	15.5 ± 2.3	0.12
*Age range*	9–18	10–18	9–18	
*Gender*				
Female	44 (75.9)	17 (89.5)	61	0.33
Male	14 (24.1)	2 (10.5)	16	
*Residence*				
Rural	6 (10.9)	1 (5.3)	7	0.67
Urban	49 (89.1)	18 (94.7)	61	
*Endoscopic diagnosis*				
Normal stomach	7 (2.5)	—	7	0.18
Endoscopic lesion	51 (97.5)	19 (100)	70	
*Eradication therapy*				
Yes	—	5 (41.7)	5	—
No	—	7 (58.3)	7	
*After therapy*				
Eradicated	—	2 (66.6)	2	—
Not eradicated	—	1 (33.3)	1	—

*N* = sample number (subjects without information in the medical records were excluded from the analysis).

**Table 4:** Endoscopy classification of gastritis according to location, intensity, and category in *H. pylori*− and *H. pylori*+ children and adolescents.

Classification	*H. pylori*		Total	*P* value
	Negative	Positive		
*N (%)*	51 (72.8)	19 (27.2)	70	
*Location*				
Antral	20 (58.8%)	3 (33.3%)	23	0.26
Pangastritis	14 (41.2%)	6 (66.7%)	20	
*Intensity*				
Mild	23 (63.9%)	5 (33.3%)	28	0.08
Moderate	12 (33.3%)	10 (66.7%)	17	
Severe	1 (2.8%)	—	1	
*Category*				
Enanthematous	24 (75%)	13 (86.7%)	37	0.46
Erosive	8 (25%)	2 (13.3%)	10	

**Table 5:** Prevalence of *Helicobacter pylori* in different studies.

Country city	Period (year)	Number of participants	Mean age–years (range)	Methods to diagnoses Hp	Local hospital-based/community-based	Prevalence of Hp (%)	Ref.
*Brazil*							
São José Rio Preto	2009/ 2012	2406	47.5 ± 16.1	Urease	Public hospital	35.4	Current study
			15.2 ± 2.3			24.7	
São Paulo	^∗^	326	6.82 ± 4.07	Serological	São Paulo Hospital	35.6	15
São Paulo	2004	1406	<10 to >60	Urease	9 of July hospital	19.3	20
	2014	1130				14.1	
Belo Horizonte	2007 to 2011	311	10.7 ± 3.3 (3 to 16)	Culture/urease/carbolfuchsin	^∗^	37.6	23
Santiago/Chile						31.4	
London/UK						7.4	
Salvador	1997 to 2003, 2005	1104	6.8 ± 0.5 (4 to 11)	Serological	Community	28.7	21
Fortaleza	2000 to 2001	610	1 to 80	^13^C-urea breath test	Community	62.9	14
*Africa*							
Ghana	2014 to 2015	240	10.5 ± 2.7 (5 to 16)	Immunochromatographic assay (fecal)	Rural community	14.2	25
*Taiwan*							
Lanyu Island	2008	796	45 ± 13.2 (12 to 89)	^13^C-urea breath test	Community	72.1	19
*Japan*							
7 geographic areas	1997 to 2013	11,470	^∗^	Serological/urinary or stool	Health center/clinic	37.6/women 43.2/ men	26
*Canada*							
Aklavik	2008	194	40.3 ± 17.1 (10 to 80)	Histology (Giemsa)	Community	66	28
*USA*							
Population study	1988 to 1994	7465	20 to 70	Serological	Community	32.5	11

^∗^ = data not available; Hp = *Helicobacter pylori*.

## Data Availability

The manuscript did not use the data generated by the public databases. All data of the patients were collected from the medical records of Hospital João Paulo II. All this information was stored in an Excel database, using codes without identifying the individuals, and they are stored under the custody of the responsible researcher (AES).
