# Erratum to “Epidemiological and Clinical-Pathological Aspects of *Helicobacter pylori* Infection in Brazilian Children and Adults”

**DOI:** 10.1155/2019/5632935

**Published:** 2019-05-02

**Authors:** Evelyn Pedroso Toscano, Fernanda Fernandez Madeira, Mayra Pinheiro Dutra-Rulli, Luiz Otavio Maia Gonçalves, Marcela Alcântara Proença, Viviane Silva Borghi, Aline Cristina Targa Cadamuro, Giselda Warick Mazzale, Ricardo Acayaba, Ana Elizabete Silva

**Affiliations:** ^1^UNESP-São Paulo State University, Department of Biology, São José do Rio Preto, SP, Brazil; ^2^UFMS-Federal University of Mato Grosso do Sul, School of Medicine, MS, Brazil; ^3^João Paulo II Hospital, Service of Endoscopy, São José do Rio Preto, SP, Brazil

In the article titled “Epidemiological and Clinical-Pathological Aspects of *Helicobacter pylori* Infection in Brazilian Children and Adults [1],” the name of the second author was given incorrectly as Fernanda Fernandes Madeira. It should have been written as Fernanda Fernandez Madeira. The revised authors' list is shown above.
